# A South American Mouse Morbillivirus Provides Insight into a Clade of Rodent-Borne Morbilliviruses

**DOI:** 10.3390/v14112403

**Published:** 2022-10-29

**Authors:** Humberto J. Debat

**Affiliations:** 1 Instituto de Patología Vegetal, Centro de Investigaciones Agropecuarias, Instituto Nacional de Tecnología Agropecuaria (IPAVE-CIAP-INTA), Córdoba X5020ICA, Argentina; debat.humberto@inta.gob.ar; Tel./Fax: +54-9-351-4973636; 2 Unidad de Fitopatología y Modelización Agrícola (UFYMA), Consejo Nacional de Investigaciones Científicas y Técnicas (CONICET), Córdoba X5020ICA, Argentina

**Keywords:** *Morbillivirus*, virus discovery, *Abrothrix*, mouse morbillivirus, Ratón oliváceo morbillivirus

## Abstract

Morbilliviruses are negative-sense single-stranded monosegmented RNA viruses in the family *Paramyxoviridae* (order *Mononegavirales*). Morbilliviruses infect diverse mammals including humans, dogs, cats, small ruminants, seals, and cetaceans, which serve as natural hosts. Here, I report the identification and characterization of novel viruses detected in public RNAseq datasets of South American long-haired and olive field mice. The divergent viruses dubbed Ratón oliváceo morbillivirus (RoMV) detected in renal samples from mice collected from Chile and Argentina are characterized by an unusually large genome including long intergenic regions and the presence of an accessory protein between the F and H genes redounding in a genome architecture consisting in 3′-N-P/V/C-M-F-hp-H-L-5′. Structural and functional annotation, genetic distance, and evolutionary insights suggest that RoMV is a member of a novel species within genus *Morbillivirus* tentatively named as South American mouse morbillivirus. Phylogenetic analysis suggests that this mouse morbillivirus is closely related to and clusters into a monophyletic group of novel rodent-borne morbilliviruses. This subclade of divergent viruses expands the host range, redefines the genomic organization and provides insights on the evolutionary history of genus *Morbillivirus*.

## 1. Introduction

The genus *Morbillivirus* encompasses viruses in the family *Paramyxoviridae* (order *Mononegavirales*) affecting not only humans but also dogs, cats, cattle and buffalos, small ruminants, seals, and cetaceans, which serve as natural hosts. Morbillivirus infection may cause diseases such as acute febrile respiratory tract infection in some animals and measles, a highly contagious infectious disease in humans that triggers severe immunosuppression, which generates over 140,000 vaccine-preventable deaths per year worldwide, mostly in sub-Saharan Africa [1] The etiological cause of measles is the measles virus (MeV) from the species *Measles morbillivirus*, one of the seven species within the genus.

Morbilliviruses are enveloped with spherical virions harboring a single-stranded, negative-sense, monosegmented RNA genome. The envelope displays two glycoproteins with receptor attachment (haemagglutinin, H) and fusion functions (F). A matrix protein (M) is associated with the inner face of the envelope. The RNA genome is protected in a ribonucleoprotein core containing a nucleocapsid protein (N), a polymerase-associated co-factor protein (P) and a large protein (L), which is an RNA-directed RNA polymerase also involved in capping and cap methylation. Morbilliviruses have a genome ranging from 15,690 to 16,050 nt, with a canonical genomic architecture encoding eight proteins in the order 3′-N-P/V/C-M-F-H-L-5′ [2]. The Morbillivirus genome organization includes a transcription unit with RNA editing encoding the phosphoprotein (P), a Zn^2+^-binding cysteine-rich protein (V) that is the edited mRNA form (one “G” added), and the overlapped non-structural protein (C) derived from leaky scanning.

Although morbilliviruses are hosted by several mammals, the apparently restricted (MeV), or limited host-range of members of each species is determined by receptors that define susceptibility of that organism to infection [3]. For at least MeV, canine distemper virus (CDV), rinderpest virus (RPV), and peste des petits ruminants virus (PPRV) the main receptors are signaling lymphocytic activation molecule family member 1 (CD150, SLAMF1).

Abundant novel viruses have been identified using metagenomic approaches, revealing the complexity of an expanding virosphere [4]. The RNA molecules of these viruses are often inadvertently co-purified with host RNAs and their sequences can be characterized from transcriptome datasets. In a consensus statement report, Simmonds et al. [5] indicate that viruses that are known only from metagenomic data can and have been incorporated as bona fide viruses into the official classification of the International Committee on Taxonomy of Viruses (ICTV). Thus, the analysis of transcriptome data represents an evolving source of novel virology insights that allows the reliable identification of new viruses in hosts with no previous record of virus infections.

While there are numerous paramyxoviruses linked to rodents, intriguingly to date, despite their extended host range, there are no morbilliviruses where rodents unequivocally serve as natural main hosts. *Abrothrix hirta* and *Abrothrix olivacea* are sigmodontine rodents endemic to Chile and Argentina. Here, by analyzing public metagenomic data, I report the identification and characterization of novel viruses associated with the South American long-haired and olive field mice representing a novel virus species from an emerging clade of rodent-borne morbilliviruses.

## 2. Materials and Methods

Virus sequence detection in public RNAseq datasets was implemented by tBLASTN searches (word size 6, expect threshold 10, scoring matrix BLOSUM62) against Vertebrata (taxid:7742) using as query the N protein of MeV (NP_056918.1) at the Transcriptome Shotgun Assembly Sequence Database (https://www.ncbi.nlm.nih.gov/genbank/tsa/ (accessed on 20 July 2022)). Datasets with hits were retrieved and subjected to BLASTP searches (E-value < 1 × 10^−5^) using as query a collection of refseq Morbillivirus proteins available at https://www.ncbi.nlm.nih.gov/protein/?term=txid11229[Organism:exp] (accessed on 20 July 2022). The obtained virus-like contigswere curated by iterative cycles of mapping of the corresponding library reads (NCBI-SRA) using Bowtie2 http://bowtie-bio.sourceforge.net/bowtie2/index.shtml (accessed on 20 July 2022) with standard parameters, which was also employed for mean coverage estimation and reads per million (RPMs) calculations. The extended, overlapped, and polished virus sequences were subsequently reassembled using rnaSPAdes de novo assembler available at the Galaxy platform https://usegalaxy.eu/ (accessed on 20 July 2022) with standard parameters. The curated sequences were inspected in detail in the Geneious 8.1.9 software platform.

To characterize thoroughly the morbillivirus sequence, virus annotation was implemented as reported elsewhere [6,7,8]. In brief, virus open reading frames (ORFs) were predicted with ORFfinder (https://www.ncbi.nlm.nih.gov/orffinder/ (accessed on 20 July 2022)) domains presence and architecture of predicted proteins was determined by the NCBI Conserved domain database v3.16 (https://www.ncbi.nlm.nih.gov/Structure/cdd/wrpsb.cgi (accessed on 20 July 2022)), HHPred/HHBlits available at https://toolkit.tuebingen.mpg.de/#/tools/ (accessed on 20 July 2022) and HHMER, http://hmmer.org/ (accessed on 20 July 2022). Secondary protein structure was predicted with Garnier http://emboss.sourceforge.net/apps/release/6.6/emboss/apps/garnier.html (accessed on 20 July 2022), signal peptides were assessed with SingnalP v5 https://services.healthtech.dtu.dk/service.php?SignalP-5.0 (accessed on 20 July 2022) and prediction of transmembrane topology with Phobius https://phobius.sbc.su.se/ (accessed on 20 July 2022). Three-dimensional modeling was implemented using the Swiss-Model platform available at https://swissmodel.expasy.org/ (accessed on 20 July 2022) with standard parameters using as best fit template the 3zdo.1.B MeV phosphoprotein [9]. Solved structures were visualized in the Chimera 1.6 software available at https://www.cgl.ucsf.edu/chimera/ (accessed on 20 July 2022). Putative contact surfaces of morbillivirus RBPs and their cognate CD150 receptors were assessed by multiple alignments using MAFTT 7.490 https://mafft.cbrc.jp/alignment/software/ (accessed on 20 July 2022) based on the predictions of Ikegame et al. [10].

Phylogenetic insights were based on the predicted replicase L protein aa alignments of recognized members of the family Paramyxoviridae provided as a resource of ICTV available at https://talk.ictvonline.org/ictv-reports/ictv_online_report/negative-sense-rna-viruses/w/paramyxoviridae/1197/resources-paramyxoviridae (accessed on 20 July 2022). The reference alignment was retrieved and a consensus alignment was generated using ClustalW with gap generation penalties of five and extension penalties of one in both multi- and pairwise alignments including RoMV, Longquan Berylmys bowersi morbillivirus 1 (LBbMV), and Wufeng Niviventer fulvescens morbillivirus 1 (WNfMV). Additionally, predicted protein N, P, M, F, and H alignments were generated by MAFTT 7.490 (BLOSUM62 scoring matrix) using as best-fit algorithm E-INS-i (M and P) or G-INS-i (N, F and H).The aligned proteins were used as input for FastTree 2.1.11 (http://www.microbesonline.org/fasttree/ (accessed on 20 July 2022)) maximum likelihood phylogenetic trees (best-fit model = JTT-Jones-Taylor-Thorton with single rate of evolution for each site = CAT) computing local support values with the Shimodaira–Hasegawa test (SH) and 1000 tree resamples.

## 3. Results and Discussion

### 3.1. Detection of Viral Sequences in RNAseq Datasets of South American Long-Haired Mice and Olive Field Mice

In order to expand our knowledge on morbillivirus diversity, I assessed an essential resource of public high-throughput sequencing RNA data available at NCBI: the Transcriptome Shotgun Assembly (TSA) Database available at https://www.ncbi.nlm.nih.gov/Traces/wgs/?view=TSA (accessed on 20 July 2022) including 866 RNAseq datasets on diverse vertebrates. In tBLASTN searches against Vertebrata (taxid:7742) using as query the N protein of MeV (NP_056918.1), I retrieved a significant hit (E-value = 1 × 10^−142^, 52.82% identity) corresponding to a 1236 nt transcript from a TSA (GenBank: GCHM00000000.1) of a renal gene expression dataset of the South American long-haired mouse, *Abrothrix hirta*, collected in natural populations (BioProject PRJNA256304 [11]). *A. hirta* is a sigmodontine rodent widely distributed in the southern area of South America occurring on both sides of the Andes, from the Chilean Region of Maule (ca. 35° S) to the Argentinean Tierra del Fuego (ca. 52° S). This specific library corresponded to half of a kidney sample from an adult male (specimen PPA528) captured using Sherman live traps, three kilometers west of Lago Pueyrredón in Río Chico department, Santa Cruz province, Argentina (47.42105° S, 71.958233° W) during field trips conducted during fall season (April 2011 and March 2012) (Supplementary Figure S1).

Further inspection by BLASTP searches (E-value < 1 ×10^−5^) of the GCHM00000000.1 TSA library using as query MeV proteins retrieved four additional transcripts ranging from 462 to 1922 ntthat showed significant hits (E-value 3.43 × 10^−30^ to 5.52 × 10^−134^, identity 34.6% to 56%) to MeV-encoded P, M, and F proteins. The tentative virus contigs were curated by iterative mapping of the corresponding 76,503,290 library reads (NCBI-SRA: SRX663121) using Bowtie2 with standard parameters. The transcripts, extended, overlapped, and polished by iterative cycles of mapping of raw reads, were subsequently reassembled into a 9941 nt-long virus sequence including a continuum from a partial 3′ leader sequence followed by N-P/V/C-M-F-hp-H- and a few short partial sequences of L (1720 nt long), with a mean coverage of 14.8x obtained with 1779 virus-derived 85 × 2 nt-long reads.

As this bio project collected a total of 16 adults of South American long-haired mice from Chile and Argentina, when assessing the read mapping with Bowtie2 using as query the assembled virus sequence of the 15 additional libraries, virus reads (82 and 62 total reads) were found in two. The samples where virus reads were detected (v+) were specimen PPA357 (SRA:SRX663109) and specimen GD1454 (SRA:SRX663075). PPA357 and GD1454 were a female from the very same location (47.42105° S, 71.958233° W) and a male collected 65 km to the west in Aysén, Chile, (47.49671666° S, 72.80861666° W), respectively. While the number of reads was certainly low, inspection and mapping of virus reads from these additional libraries to the PPA528 consensus revealed some fixed SNPs among libraries (Supplementary Figure S2) suggesting: (*i*) on the one hand that the reads were evidence of apparently three distinctive virus isolates, while (*ii*) on the other hand ruling out that these few reads corresponded to spillover from the PPA528 sample or contamination artifacts from index-hopping during library processing.

In order to expand the survey of this virus to additional hosts, all available RNAseq datasets of rodent subfamily *Sigmodontinae* (*Cricetidae*), including New World rats and mice with at least 376 species, were retrieved from NCBI. Of the 79 additional publicly available transcriptome datasets of mice, including members of the *Sigmodon*, *Oligoryzomys*, and *Abrothrix* genera (Supplementary Table S1), virus reads were detected by mapping using Bowtie2 in two libraries of *Abrothrix olivacea* (Supplementary Figure S1). The olive field mouse (*A. olivacea*) is the rodent that shows the broadest geographic distribution in the area of southern South America. It ranges from the northernmost region of Chile (ca. 18° S) to central-western Argentina (ca. 35° S) and towards Patagonia, where it reaches the south of Tierra del Fuego (ca. 56° S).In elevation, it is found from sea level to up to 2500 m of altitude [12]. Regarding viruses and abrotrichine rodents, to my knowledge, there are no reports oriented to the detection or characterizations of viruses linked to these mice. It is worth mentioning the detection of Andes hantavirus virus-reactive antibodies in *A. olivacea* exemplars from southern Chile [13], and that experimental conditions have indicated that the olive field mouse is susceptible to hantavirus infection [14].

The two specific v+ libraries corresponded to kidney samples from an adult male (specimen PPA444, SRA: SRX4099316) also captured in the Río Chico department, Santa Cruz province, Argentina, but 265 km southeast of the location where PPA528 and PPA357 were collected (49.42105° S, 69.958233° W). The other sample, also an adult male (specimen GD1411, SRA: SRX4099309), was captured 870 km to the northeast, in Fundo San Martín, Región de Los Ríos, Chile (39.649233° S, 73.19255° W); both GD1411 and PPA444 were collected in the same study (BioProject PRJNA471316 [15]). With iterative cycles of relaxed mapping (Bowtie2 parameters-very-sensitive-local) of SRX4099309 raw reads, extension and subsequent reassembly, a 9948 nt-long virus sequence from the GD1411 sample was obtained, including a continuum from a partial 3′ leader sequence to N-P/V/C-M-F-hp-H- and a few short partial sequences of L (2736 ntlong), with a robust mean coverage of 128x obtained with 12,630 virus-derived 101 × 2 nt-long reads. Notably, implementing the same pipeline to sample PPA444 employing the SRX4099316 library a coding-complete virus sequence with the genome architecture 3′-N-P/V/C-M-F-hp-H-L-5′ was assembled corresponding to 16,568 nt supported by a 17.7x mean coverage from 2897 virus-derived 101 × 2 nt-long reads.

A rapid comparison based on sequence alignments of the three consensus virus sequences (Supplementary Figure S3) indicated that while divergent, the % identity of predicted proteins ranged between 88.2 and 99.8%, suggesting that the sequences corresponded to three distinctive strains of the same virus which I tentatively dubbed Ratón oliváceo morbillivirus (RoMV). Indetail, the viruses assembled from the PPA444 and PPA528 samples are highly similar, with their ORFs and predicted proteins sharing over a 99% sequence identity. In contrast, the GD1411 virus is clearly more divergent, sharing a lower 85–89% nt identity and 88–98% aa identity of the predicted proteins, the P and H proteinsthe more distinctive and N and M the more similar, indicating the significant preeminence of synonymous mutations on the GD1411 virus. Perhaps it is worth emphasizing that the GD1411 mouse was collected on the other side of the Andes mountain range, over 850 km and more than 1100 km northeast of the places where PPA528 and PPA444 were captured, signifying that geographical isolation could provide some clues to the evolutionary history of these viruses. The significant diversity revealed by these three mouse viruses could indicate a long-lasting virus–host relationship between RoMV and abrotrichine rodents. In turn, the consensus assembly from sample PPA444 that comprised a complete coding, and (near) complete genome, was used as reference for structural and functional annotation, genomic comparison and evolutionary insights into RoMV.

### 3.2. Characterization of a Novel Virus by In Silico Analysis

**Figure 1:**
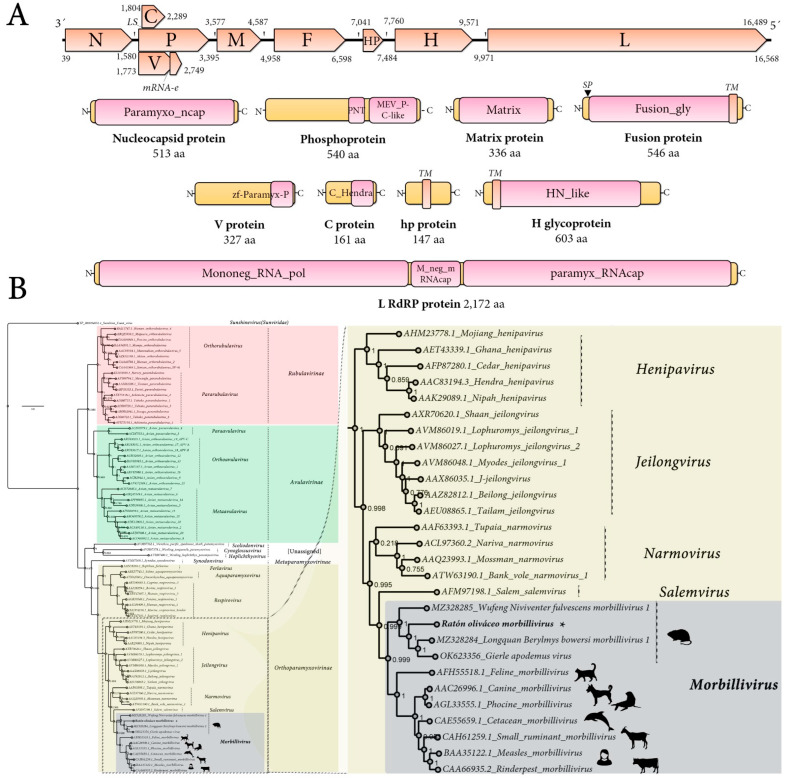
Genomic architecture and phylogenetic insights of Ratón oliváceo morbillivirus. (**A**) Genome graphs depicting architecture and predicted gene products of RoMV. The predicted coding sequences are shown in orange arrow rectangles; start and end coordinates are indicated. Gene products are depicted in curved yellow rectangles and size in aa and name are indicated below. Predicted domains or HHP red best-hit regions are shown in curved pink rectangles. Abbreviations: N, nucleoprotein CDS; P, phosphoprotein CDS; M, Matrix protein CDS; F, Fusion protein CDS; HP, hypothetical protein CDS; H, Hemagglutinin glycoprotein CDS; L, RNA-dependent RNA-polymerase CDS; TM, trans-membrane domain; SP, signal peptide; LS, leaky scanning; mRNA-e, RNA editing. Domain abbreviations are described in the main text. (**B**) Maximum likelihood phylogenetic tree based on amino acid alignments of the L polymerase of RoMV and members of accepted species within family *Paramyxoviridae*. The right panel represents an inset of the complete phylogenetic tree that is available as Supplementary Figure S9. The tree is rooted at Sunshine coast virus (family *Sunviridae*). The scale bar indicates the number of substitutions per site. Node labels indicate FastTree support values. Silhouettes represent natural hosts. Genbank accession numbers of the proteins used are depicted before each virus species name. * indicates RoMV.

The genome organization of the tentatively named Ratón oliváceo morbillivirus is characterized by a ≈ 16,658 nt-long negative-sense single-stranded RNA containing six main ORFs in the anti-genome, positive-sense orientation. In addition, the second ORF includes a transcription unit with RNA editing and an overlapping ORF (P/V/C) and between the F and H genes there is an additional accessory ORF. In sum, the genomic architecture of theRoMV is 3′-N-P/V/C-M-F-hp-H-L-5′ (Figure 1A). As expected for paramyxoviruses, the genes are separated by intergenic gene junction regions, composed of the polyadenylation signal of the preceding gene, a short intergenic region, and the transcriptional start of the following gene [2]. The detected consensus gene junction region of the RoMV is consistent with morbilliviruses that have a conserved intergenic motif (CUU) between the gene-end and gene-start of adjacent genes following the structure “AAAA-CUU-AGG” (Table 1).

BLASTP searches of predicted products (Table 2) tentatively identified these ORFs as potentially encoding: a nucleocapsid protein (N; 513 aa), a phosphoprotein (P; 540 aa), a V non-structural protein (V; 325 aa), a C non-structural protein (C; 161 aa), a matrix protein (M; 336 aa), a fusion protein (F; 546 aa), a small hypothetical protein (hp; 147 aa), a Hemagglutinin glycoprotein (H; 603 aa), and an RNA-dependent RNA polymerase (L; 2172 aa). Importantly, all best hits based on highest sequence identity scores, which ranged between 25.1% (H) and 63.7% (M), were morbilliviruses, more specifically MeV, Longquan Berylmys bowersi morbillivirus 1 (LBbMV), and Wufeng Niviventer fulvescens morbillivirus 1 (WNfMV). LBbMV and WNfMV corresponded to recently released virus sequences, which are as yet unpublished and have been annotated as unclassified morbilliviruses. The metadata of their GenBank accessions indicated that LBbMV (GenBank accession no. MZ328284) was identified in the Bower’s white-toothed rat (*Berylmys bowersi*), a rodent from the family *Muridae* that is native to Southeast Asia.WNfMV (GenBank accession no. MZ328285) was detected in the chestnut white-bellied rat (*Niviventer fulvescens*), another rodent from the family *Muridae*.

Structural and functional annotation indicates that the 513 aa RoMV-N protein harbors a paramyxovirus nucleocapsid protein domain (Paramyxo_ncap, E-value = 0, coordinates 1–512) that is involved in tightly encapsidating the viral RNA and interacting with several other viral-encoded proteins, all of which are involved in controlling replication. RoMV-N presents the conserved MA(S,T)L motif of morbilliviruses, and appears to share the three key conserved motifs in paramyxoviruses and nuclear export signals and NLS (Supplementary Figure S4). The 540 aa P protein, which plays a crucial role by positioning L onto the N/RNA template through an interaction with the C-terminal domain of N, is a co-factor of the RdRP, includes a paramyxovirus structural protein V/P N-terminus domain (Paramyxo_PNT, pfam13825, E-value = 3.67 × 10^−3^, coordinates 274–347), and a paramyxovirus P/V phosphoprotein C-terminal domain (Paramyx_P_V_C, pfam03210, E-value = 1.13 × 10^−16^, coordinates 372–536) (Figure 1A). Most of its 540 amino acids, as expected, appear to be in a natively disordered state, and the C-terminal conserved residues are putatively folded into a three-helical bundle that binds to the C-terminal tail of N and has an oligomerization domain that forms a long tetrameric coiledcoil that is stabilized at its N terminus by a helical bundle linking protomers. Three-dimensional modeling using the Swiss-Model platform using as best fit template the 3zdo.1.B MeV phosphoprotein showed that RoMV-P forms a tetrameric coiled coil similar length (63 aa) and conserved structure but less highly packed than the measles virus P protein (Supplementary Figure S5). The 327 aacysteine-rich non-structural V protein generated by mRNA editing by incorporating an additional “G” at coordinate 2512 of what encodes the P mRNA has a zinc-binding domain of *Paramyxoviridae* V protein at its C-terminal region (zf-Paramyx-P, E-value = 2.4 × 10^−15^, coordinates 280–323). The Vprotein is generated by an A-rich context where the RNA transcriptase ‘stutters’ on the template at the editing motif that is “AAAAAGGG” in RoMV. This stuttering results in the insertion of one pseudo-templated G shifting the reading frame to access the alternative ORF V [2]. The 161 aa C protein, which is generated by leaky scanning of the P mRNA that results in the translation of an overlapped ORF 31 nt downstream of the AUG of P, presents a C protein from thehendra and measles viruses domain (C_Hendra, pfam16821, E-value = 8.10 × 10^−5^, coordinates 1–146). The C protein has been involved in host defense interaction, for instance MeV C is implicated in modulation of interferon signaling but also in pathogenicity and virulence as is the case for CDV C [16], andMeVC downregulates viral RNA synthesis and allows the virus to escape detection by the cytosolic RNA sensors and finally prevents IFN production [17]. The non-glycosylated membrane or matrix protein (M) is 336 aa long and has a viral matrix protein domain (Matrix, pfam00661, E-value = 6.34 × 10^−118^, coordinates 6–326). The M protein appears to be the most conserved protein of the RoMV, sharing 63.6% aa identity with that of LBbMV. The 546 aa F glycoprotein presents as expected a signal peptide at its N-terminal region and a transmembrane domain at its C-end (Figure 1A). The F protein functional annotation pinpoints a typical fusion glycoprotein domain (Fusion_gly, pfam00523, E-value = 1.04 × 10^−128^, coordinates 23–478). Unexpectedly, a small 147 aa protein (hp) is found in the F-H intergenic region showing no homology to any protein, nor domains. No similarities are found to motifs/domains/peptides/proteins in any database to hp when Psi-blast, HHblits, HHPred, or HMMER searches are implemented (see below for more details). The 603 aa surface H glycoprotein shows a typical haemagglutinin-neuraminidase of the paramyxoviridae domain (HN_like, cd15464, E-value = 3.09 × 10^−20^, coordinates 207–579) and a N-terminal transmembrane domain. The H protein is the most divergent encoded main protein RoMV showing only a 16–22% aa best identity with the H of LBbMV and WNfMV.

As CD150 is the tentative main receptor of morbilliviruses I used the primary data from *A. olivacea* to reconstruct the protein using as query the signaling lymphocytic activation molecule family member 1 coding sequence from the available hispid cotton rat CD150 (*Sigmodonhispidus,* JX424845), eventually generating a complete mRNA 1278nt-long encoding an *A. olivacea* 340 aa protein showing 81.5% aa identity to that of the hispid cotton rat. In order to try to glimpse the RBP-CD150 interactions that could be involved in determining host tropism, I compared the amino acid sequences at the putative contact surfaces of morbillivirus RBPs and their cognate CD150 receptors based on the predictions of Ikegame et al. [10] (Supplementary Figure S6). Alignment of putative key regions in some morbillivirus H proteins implicated in CD150 interactions showed virus-specific changes, with some residues highly conserved and others significantly variable, which may suggest the adaptation of morbillivirus H to the putative CD150 receptors of their cognate host (Supplementary Figure S6). Modeling and experimental assessment of these in silico predictions could shed some light on the specific role of *A. olivacea* CD150 in the host interaction and range of the RoMV. Finally, the 2172 aa long L protein presents a Mononegavirales RNA-dependent RNA-polymerase domain (Mononeg_RNA_pol, pfam00946, E-value = O, coordinates 16–1107) followed by a *Paramyxoviridae* family mRNA-capping enzyme region (paramyx_RNAcap, TIGR04198, E-value = 2.96 × 10^−176^, coordinates 1224–2172) including a mRNA (guanine-7-)methyltransferase (G-7-MTase) (G-7-MTase, pfam12803, E-value = 1.74 × 10^−87^, coordinates 1483–1793) that catalyzes cap methylation.

The genome of RoMV presents some peculiarities that distinguish it from other assigned morbilliviruses. For instance, the (nearly) complete sequence with 16,658 nt represents to date the lengthiest morbillivirus reported (Supplementary Figure S7**)**, being as it is at least 518 nt longer than the Feline morbillivirus (FeMV), which is characterized for a long M-F intergenic region and 5′ trailer sequence. The longer nature of RoMV is not explained by its coding regions, which are of typical size, but by the longest intergenic regions described yet within the genus (Supplementary Table S2). The RoMV presents the lengthiest N-P, P-M, and F-H intergenic regions reported yet. In the latter, the presence of an accessory putative ORF encoding a 147 aa hypothetical protein with a transmembrane domain is not a hallmark of morbilliviruses. For instance, as other examples, the mumps virus (*Orthorubulavirus*) presents a small hydrophobic (SH) protein gene encoded between F and H [18]. It is worth noting that the rodent putative morbillivirus WNfMV shares, in the same genomic context between F and H, an ORF encoding a small 74 aa protein also with a transmembrane domain. While it is tempting to consider that these accessory proteins may have some role in rodent–host interaction, the absence of this ORF in LBbMV hampers any conclusion, its function remains elusive, and its presence is not a distinguishing feature of this subclade. A short integral membrane protein (SH) and/or transmembrane protein (tM) located between F and H is not exceptional in paramyxoviruses and can be found for instance in some members of the subfamily *Orthoparamyxovirinae* such as rodent viruses from genus *Jeilonvirus* where it is thought to be involved in cell-to-cell fusion [19]. Besides genomic location, relative size, and presence of a transmembrane signal, there is no apparent identity or reminiscence of homology between these jeilonvirus proteins and the ones from RoMV and WNfMV; thus, I decided to dub it as hypothetical protein (hp) instead of SH or tM to avoid confusion (Supplementary Figure S8). Both RoMV and LBbMValso include a significantly long H-L intergenic region of about three times the typical size in morbilliviruses mainly derived from an unusually long AU-rich (65–70%) H mRNA 3′UTR.

### 3.3. Phylogenetic Analysis of a Novel Virus

Phylogenetic insights based on the predicted replicase of RoMV were employed to assess the putative evolutionary placement of this virus. To this end, the L protein aa alignment of recognized members of the family *Paramyxoviridae* provided as a resource of ICTV available at https://talk.ictvonline.org/ictv-reports/ictv_online_report/negative-sense-rna-viruses/w/paramyxoviridae/1197/resources-paramyxoviridae (accessed on 20 July 2022), was retrieved and a consensus alignment was generated using ClustalW. The obtained paramyxovirus L tree clearly shows that RoMV clusters together with other viruses within the genus *Morbillivirus* (Figure 1B; Supplementary Figure S9). In addition, RoMV appears to have a close evolutionary relationship with WNfMV, LBbMV and a putative morbillivirus linked to the wood mouse (*Apodemus sylvaticus*), a murid rodent native to Europe [20], branching together forming a clade of rodent morbilliviruses. The recently reported “Apodemus morbillivirus” was detected in a wood mouse cadaver that had been killed by cats or vehicles, collected in Belgium and was dubbed Gierle apodemus virus (GaMV, GenBank accession no. OK623356, release date 18 May 2022). A comparison of GaMV and RoMV genomes based on sequence alignments showed a relatively low 56% nt pairwise identity and their predicted proteins ranged from 21.1% (H protein) to as high as 64.5% (M protein) pairwise identity. To further confirm the evolutionary findings based on L alignments, N, P, M, F, and H phylogenetic trees were generated using proteins from viruses of genus *Morbillivirus*, RoMV, WNfMV, LBbMV, and GaMV and the respective proteins of Tupaianarmovirus and Nariva narmovirus (genus *Narmovirus*). In all cases, unequivocally, RoMV clustered with morbillivirus forming a sub-clade with the rodent WNfMV, LBbMV, and GaMV (Supplementary Figure S10).

The ICTV species demarcation criteria of morbilliviruses are based on distance in the phylogenic tree of complete L protein based on tree topology and branch length between the nearest node and the tip of the branch. This length is defined as 0.03 in the trees generated in the ICTV paramyxovirus resource and used as input for the L tree reported here.As the branch length from the node separating RoMV/LBbMV in substitutions per site of the obtained consensus tree is well above this threshold, RoMV appears to correspond to a new virus, a putative member of a novel species within genus *Morbillivirus* that I tentatively name ‘South American mouse morbillivirus’.

## 4. Conclusions

In summary, the scrutiny of public NCBI-SRA transcriptome data constitutes a reliable source for the detection of novel viruses. By implementing a simple pipeline of RNA sequence analysis, I report the identification and molecular characterization of a novel rodent virus. The analyses revealed that these viruses are tentative members of a novel species of the genus *Morbillivirus*. The mouse morbillivirus reported here in parallel with additional recent reports and posted sequences sheds light on an emerging novel subclade of rodent morbilliviruses. More importantly, these novel viruses expand the genome architecture and host range of morbilliviruses providing new clues for the understanding of the evolutionary history of this genus of viruses.Future studies should assess additional rodent viruses, the potential prevalence of RoMV or related viruses in *Sigmodontinae* rodents, unravel whether the infection of these novel viruses is associated to specific symptoms, and explore the zoonotic and pathogenic potential of these viruses.

## Figures and Tables

**Table 1:** Predicted intergenic regions of RoMV.

Gene Region	Poly-Adenylation	Intergenic Spacer	Transcription Start	Gene Regon
3′ leader	na	na	AGGTGCCAGC	N gene
N gene	ATTAAGAAAAA	CTT	AGGACCAAAG	P gene
P gene	ACTAAAGAAAA	CTT	AGGATTTAAA	M gene
M gene	ATTCAATAAAA	CTC	AGAGAATCTA	F gene
F gene	ATTAAGAAAAA	CTT	AGGAGGTAAA	HP gene
HP gene	ACTAAAGAAAA	CTT	AGGGTTAATG	G gene
G gene	TAAAGAAAACA	CTT	AGGAATAACG	L gene
L gene	ATTAAGAAAAA	CTT	na	5′ leader
Consensus 85%	AYTAARnAAAA	CTT	AGGRnnWAnR	
Consensus 100%	WHWMRRnAAMA	CTY	AGRDnnHMnV	
Consensus MeV	VHHWHDnAAAA	CKT	AGGRnRMARG	

na, not available.

**Table 2:** Summary of RoMV genome-encoded proteins and best blasp hits.

ORF	Gene	Putative Function	Lenght (aa)	MW (kD)	IEP ^a^	BLASTP E-Value	BLASTP Ide (%)	BLASTP Score	Best Hit Abbr
1	N	Nucleocapsid protein	513	57.2	4.9	0.0	**54.8**	542	LBbMV ^b^
2	P	Phosphoprotein	540	60.2	5.1	3 × 10^−58^	**30.6**	212	LBbMV ^b^
3	V	V protein	327	36.1	5.7	1 × 10^−15^	**35.5**	87.4	MeV ^c^
4	C	C protein	161	18.8	11.1	0.037	**37.0**	44.7	BvV1 ^d,€^
5	M	Matrix protein	336	37.3	9.1	7 × 10^−154^	**63.7**	445	LBbMV ^b^
6	F	Fusion protein	546	59.9	7.4	6 × 10^−178^	**53.0**	523	LBbMV ^b^
7	HP	Hypothetical protein	147	16.8	9.6	Nh ^e^	Nh ^e^	Nh ^e^	Nh ^e^
8	H	Glycoprotein	603	66.7	7.7	5 × 10^−46^	**25.1**	182	LBbMV ^b^
9	L	RdRNA Polymerase	2172	248.7	7.1	0.0	**62.8**	2915	LBbMV ^b^

^a^ IEP, isoelectric point pH. ^b^ LBbMV, Longquan Berylmys bowersi morbillivirus 1. ^c^ MeV, Measles morbillivirus. ^d^ BvV1, bank vole virus 1. ^e^ nh, no hit. ^€^ while the best obtained blastp hit of RoMV C protein retrieved BvV1, RoMV C closest identity corresponds to the unannotated overlapped gen product of LBbMV with shares with RoMV C a 37.4% aa identity.

## Data Availability

The virus sequences assembled in this study corresponding to Ratón oliváceo morbillivirusare available in the Third Party Annotation Section of the DDBJ/ENA/GenBank databases (RoMV strains PPA444 (accession number BK061229), GD1411 (BK061230) and PPA528 (BK061231)) and can be found as Supplementary Material 1 of this article. The primary data underlying these assemblies are publicly available on NCBI-BioProjects PRJNA471316 and PRJNA256304 and SRA experiments SRX4099316, SRX4099309 and SRX663121.

## Supplementary Materials

The following supporting information can be downloaded at: https://www.mdpi.com/article/10.3390/v14112403/s1. Figure S1. South American map depicting the location (pinned) of the captured mice where RoMV was detected. Figure S2. Mapping of RoMV PPA357 and GD1454 virus reads against the PPA528 consensus assembly. Three representative regions are depicted showing apparent fixed SNPs which differentiate the emerging consensus sequence of each sample. Figure S3. Genetic distance comparison of RoMV strains. Figure S4. Multiple alignments of N proteins of RoMV and other morbilliviruses and recently described putative rodent morbilliviruses. Figure S5. Side and top views of cartoon representations of the P oligomerization domains of RoMV and measles virus (MeV) generated by the SWISS-MODEL platform using as best fit template the 3zdo.1.B phosphoprotein. Figure S6. AA sequence alignment of CD150 from some morbillivirus hosts and A. olivacea. CD150 from the 5 mammals that are the natural hosts, Mus musculus and olive field mouse aligned by clustalw. Figure S7. Genome graphs drawn at scale depicting accepted morbilliviruses, WNfMV, LBbMV and RoMV. Figure S8. Diagrams representing transmembrane topology of hypothetical protein of RoMV and WNfMV and of J-virus (jeilonvirus) SH and tM as predicted by the Phobius tool. Figure S9. Maximum likelihood phylogenetic tree based on amino acid alignments of the L polymerase of RoMV and members of accepted species within family Paramyxoviridae. Figure S10. Maximum likelihood phylogenetic tree based on amino acid alignments of the N, P, M, F, H and L proteins of RoMV and members of accepted species within genus Morbillivirus, WNfMV, LBbMV (blue rectangle) and of Tupaia narmovirus and Nariva narmovirus (genus Narmovirus) (yellow rectangles). Table S1. Data from each NCBI-SRA library assessed in this study. Table S2. General characteristics of ORF sizes of morbilliviruses.
