# Supplementary figures and images for: A South American Mouse Morbillivirus Provides Insight into a Clade of Rodent-Borne Morbilliviruses
Source: Viruses. 2022 Oct 29;14(11):2403. doi: 10.3390/v14112403 (PMC9697977; doi:10.3390/v14112403)

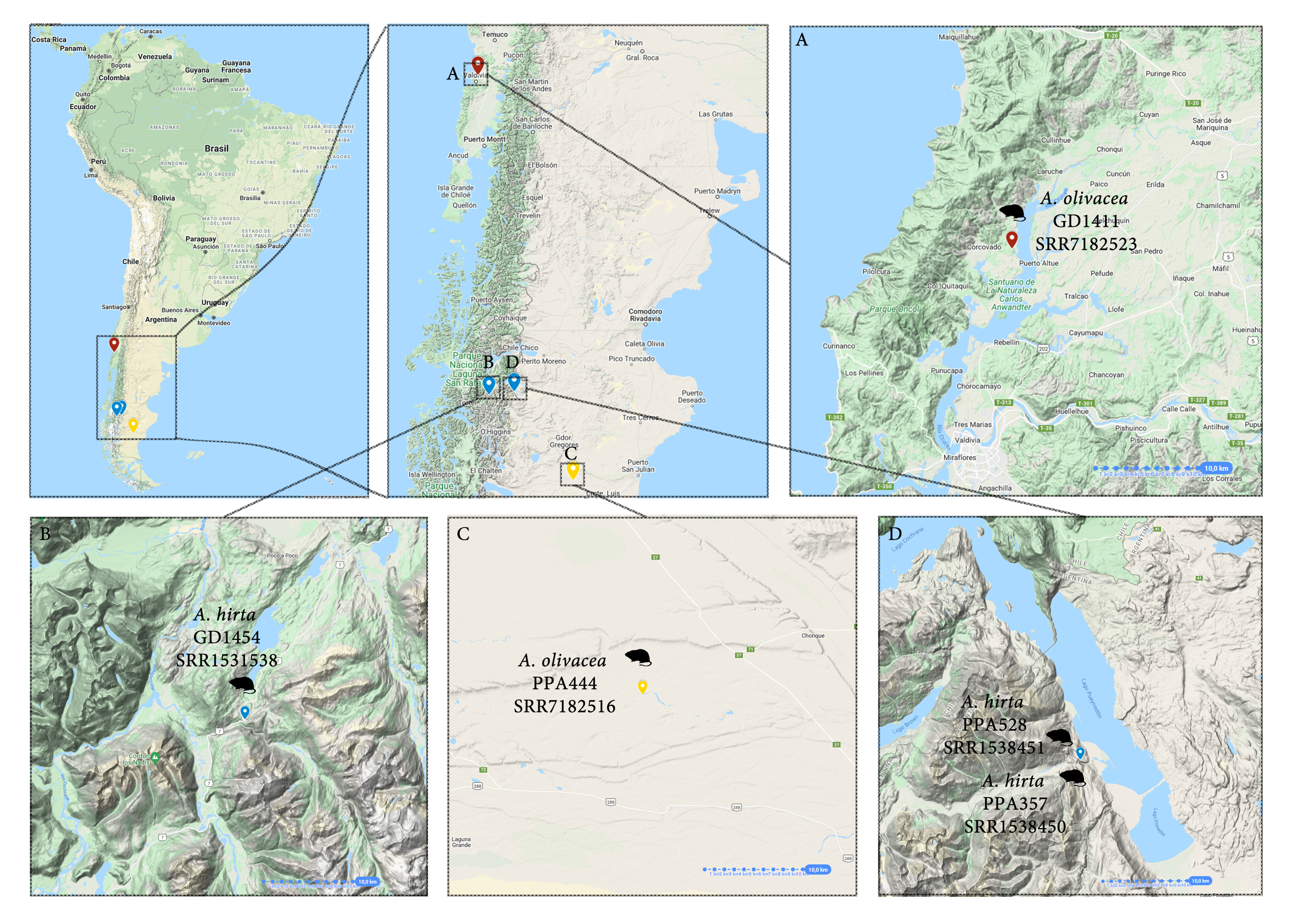

Supplement: Supplementary file 1 [file viruses-14-02403-s001.zip › Supplementary Figure S1.jpg]

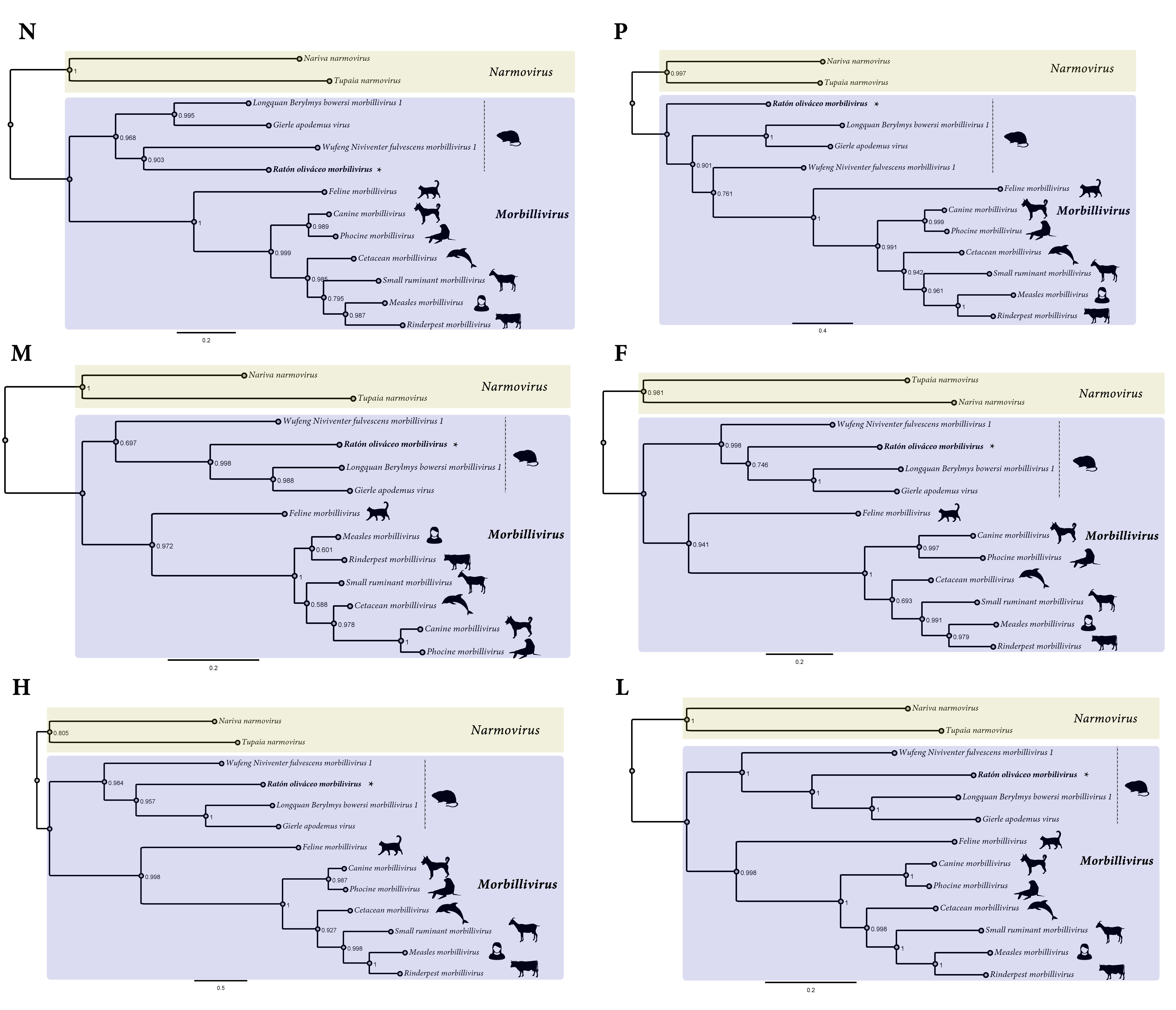

Supplement: Supplementary file 1 [file viruses-14-02403-s001.zip › Supplementary Figure S10.jpg]

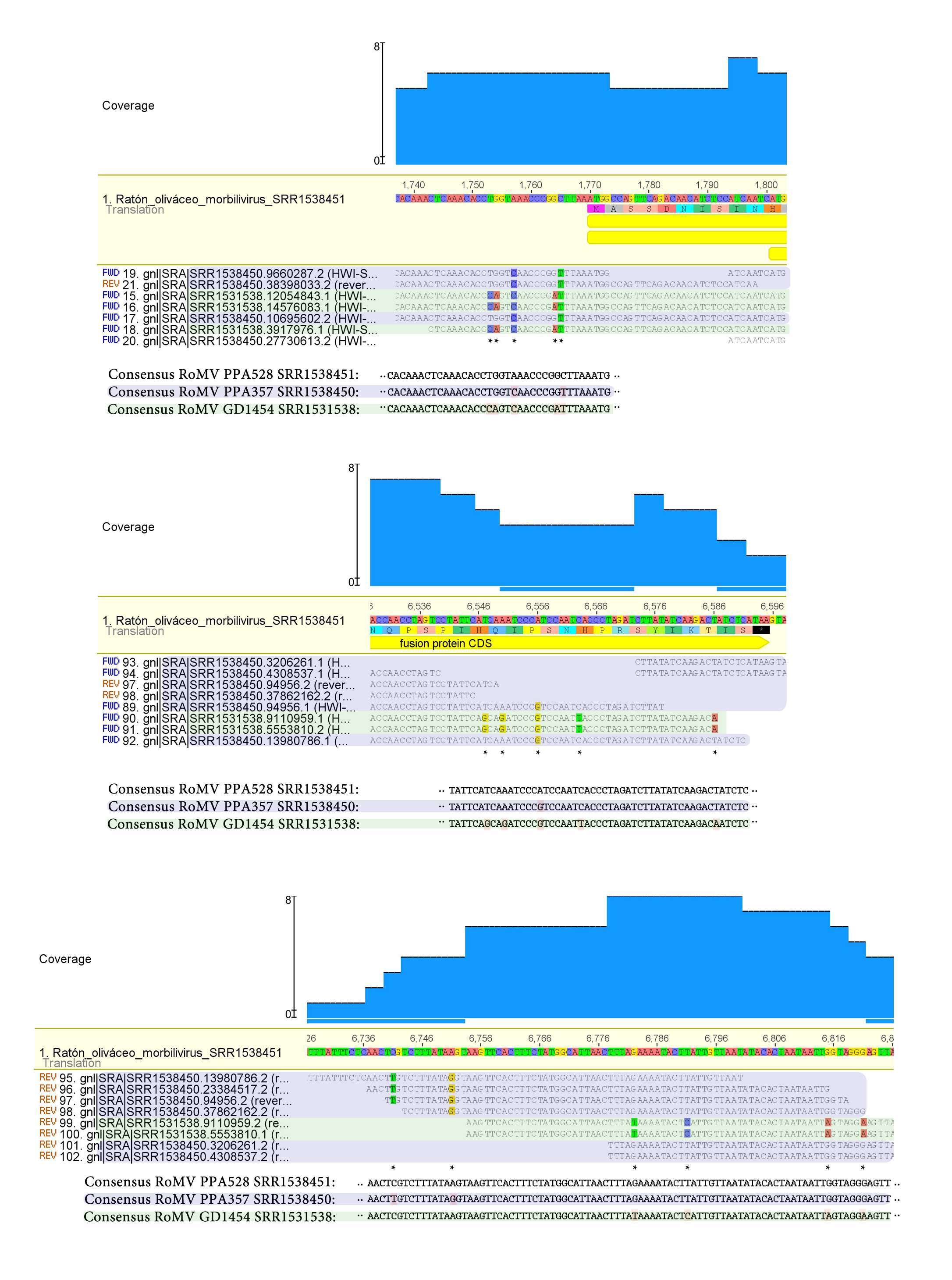

Supplement: Supplementary file 1 [file viruses-14-02403-s001.zip › Supplementary Figure S2.jpg]

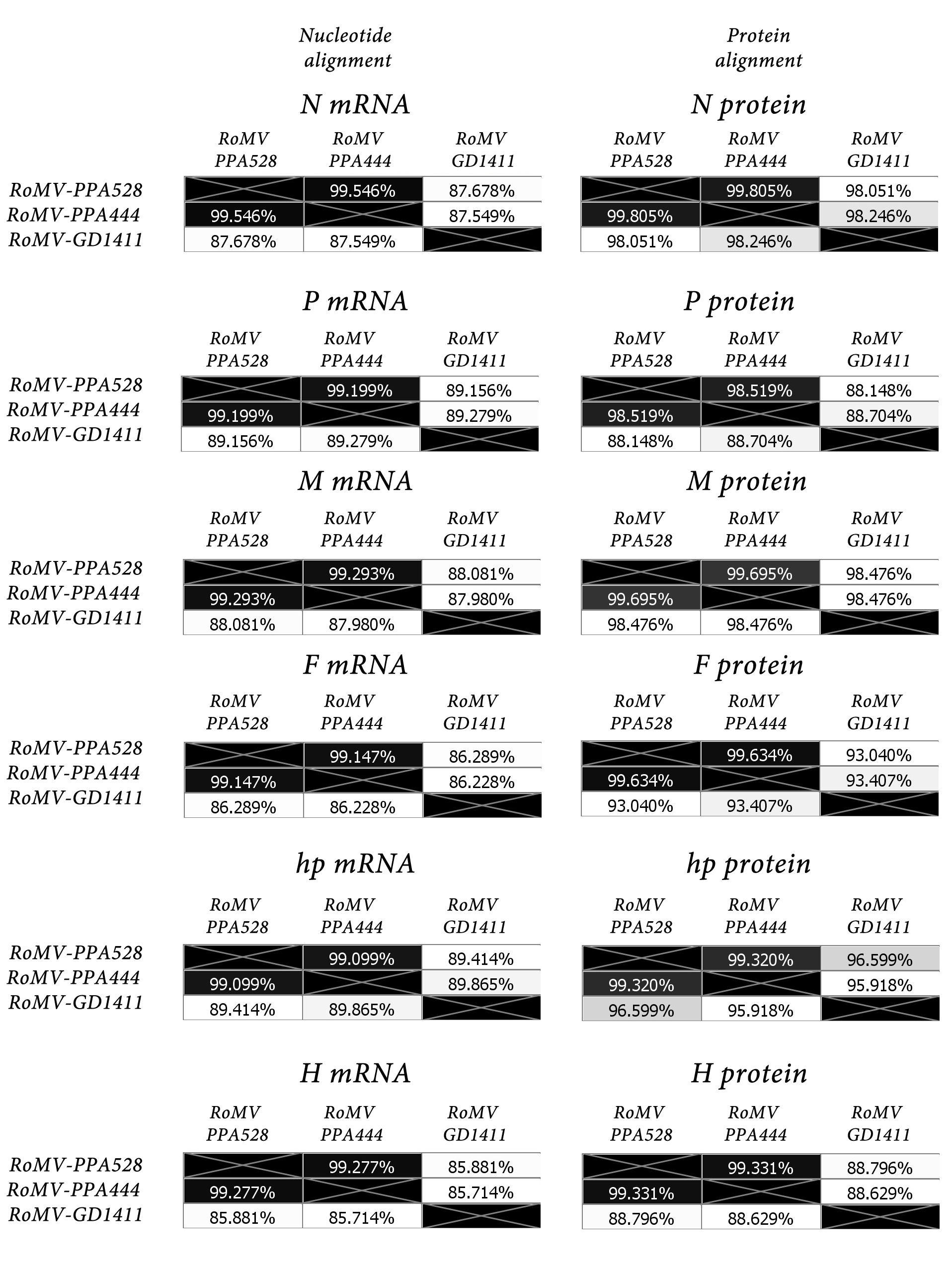

Supplement: Supplementary file 1 [file viruses-14-02403-s001.zip › Supplementary Figure S3.jpg]

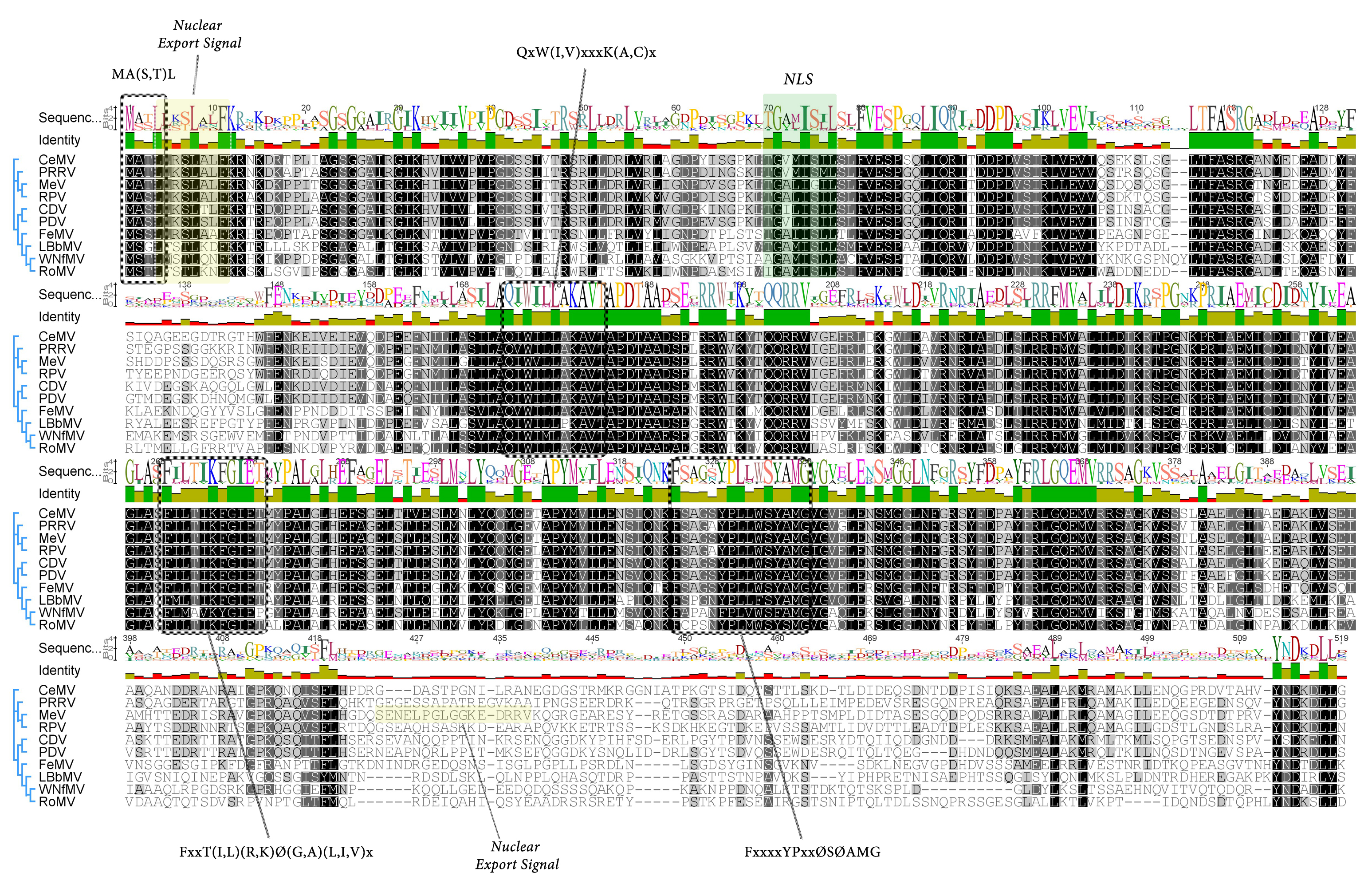

Supplement: Supplementary file 1 [file viruses-14-02403-s001.zip › Supplementary Figure S4.jpg]

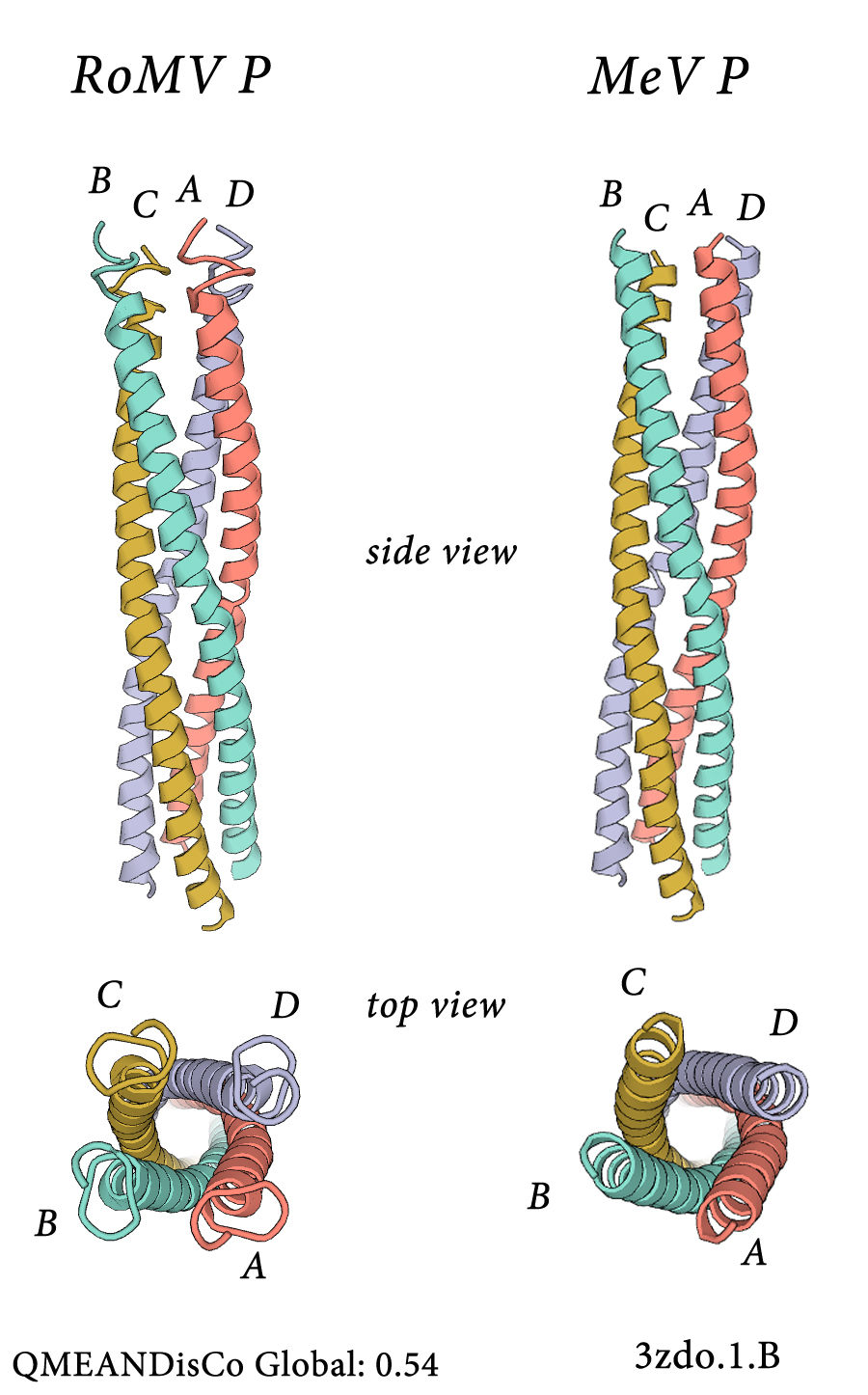

Supplement: Supplementary file 1 [file viruses-14-02403-s001.zip › Supplementary Figure S5.jpg]

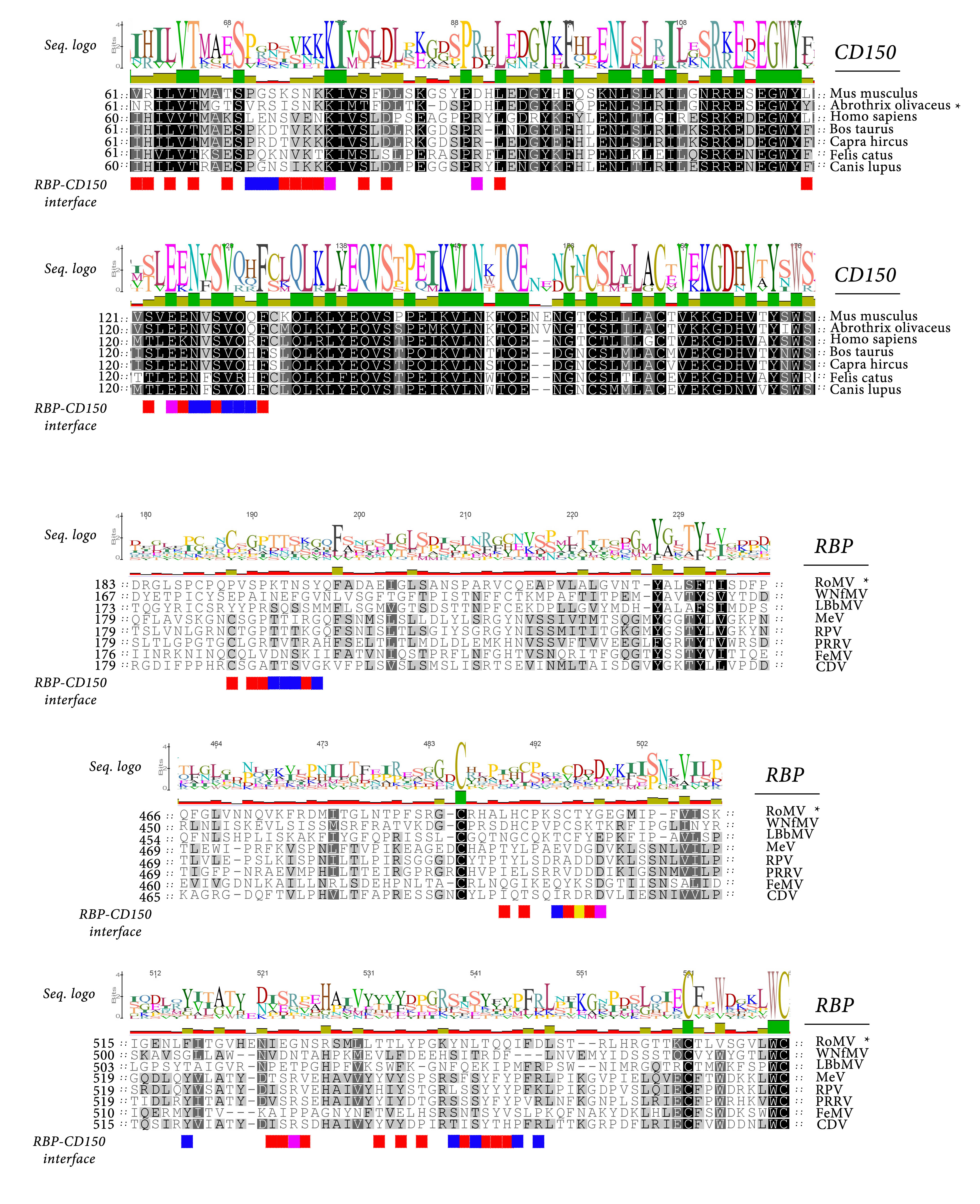

Supplement: Supplementary file 1 [file viruses-14-02403-s001.zip › Supplementary Figure S6.jpg]

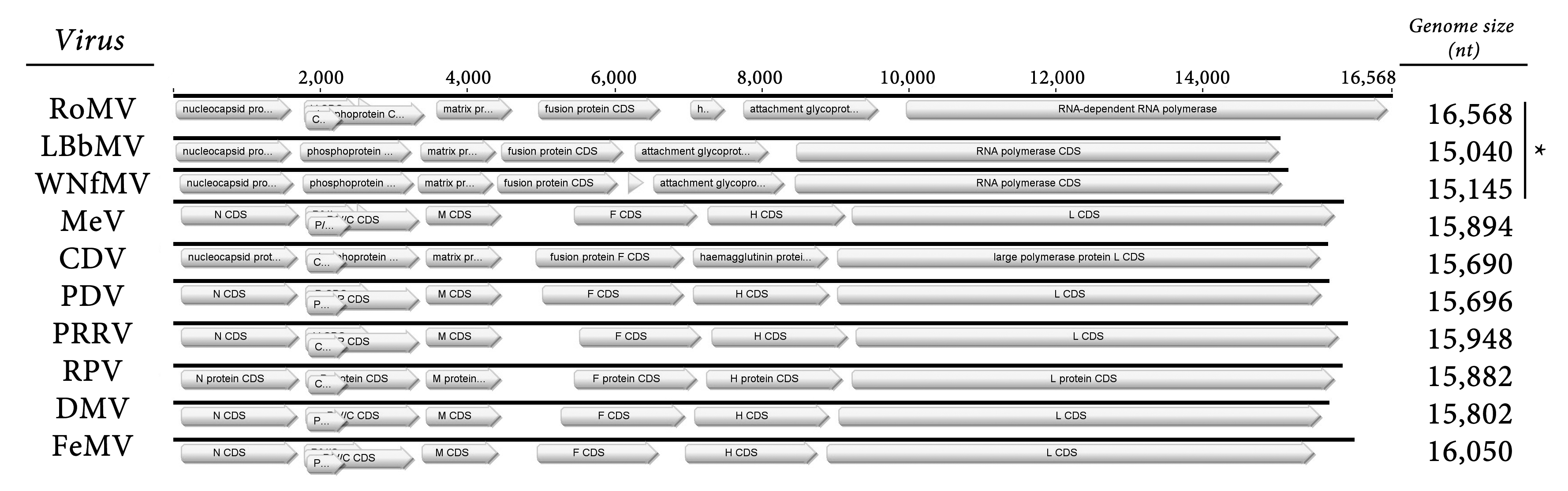

Supplement: Supplementary file 1 [file viruses-14-02403-s001.zip › Supplementary Figure S7.jpg]

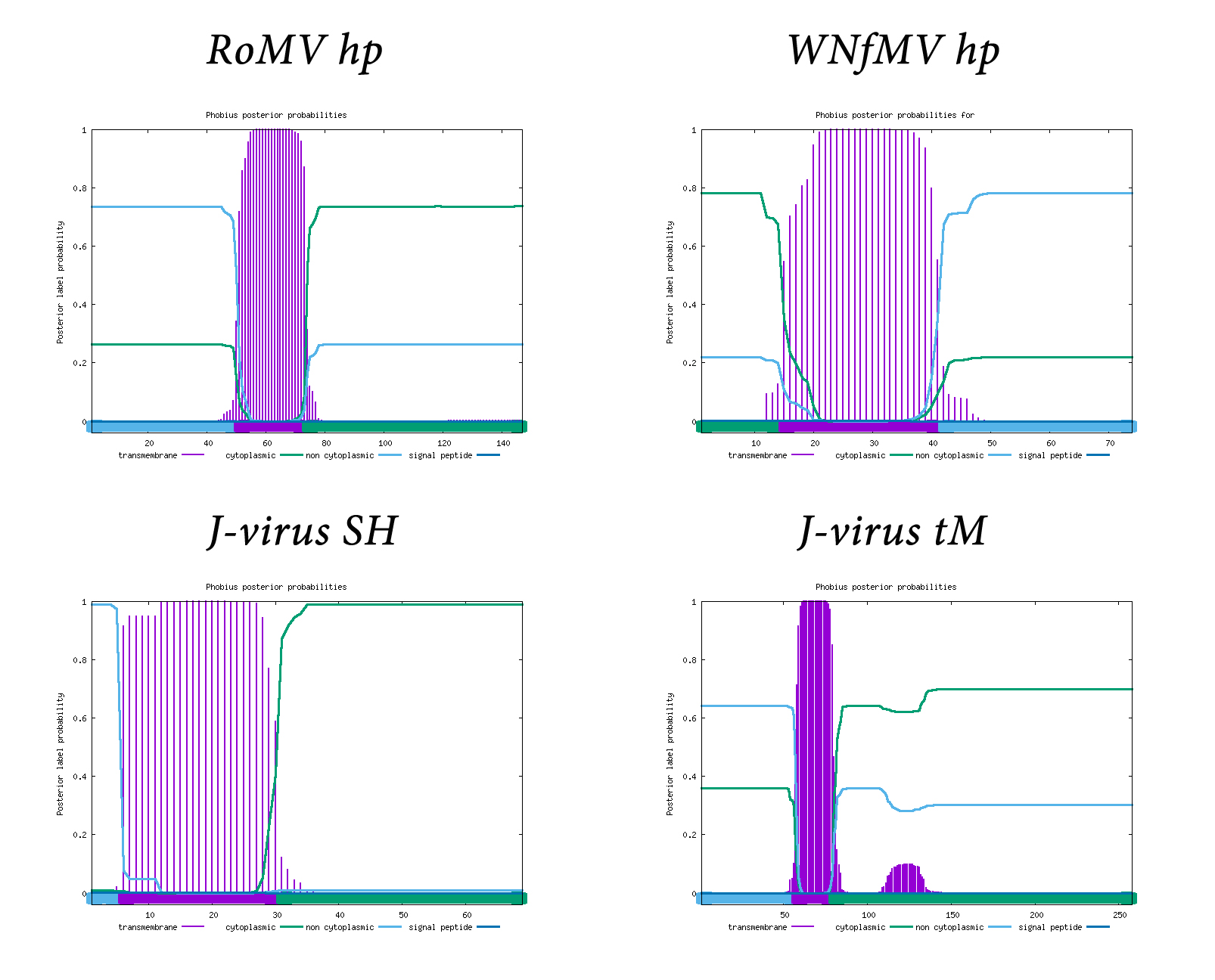

Supplement: Supplementary file 1 [file viruses-14-02403-s001.zip › Supplementary Figure S8.jpg]

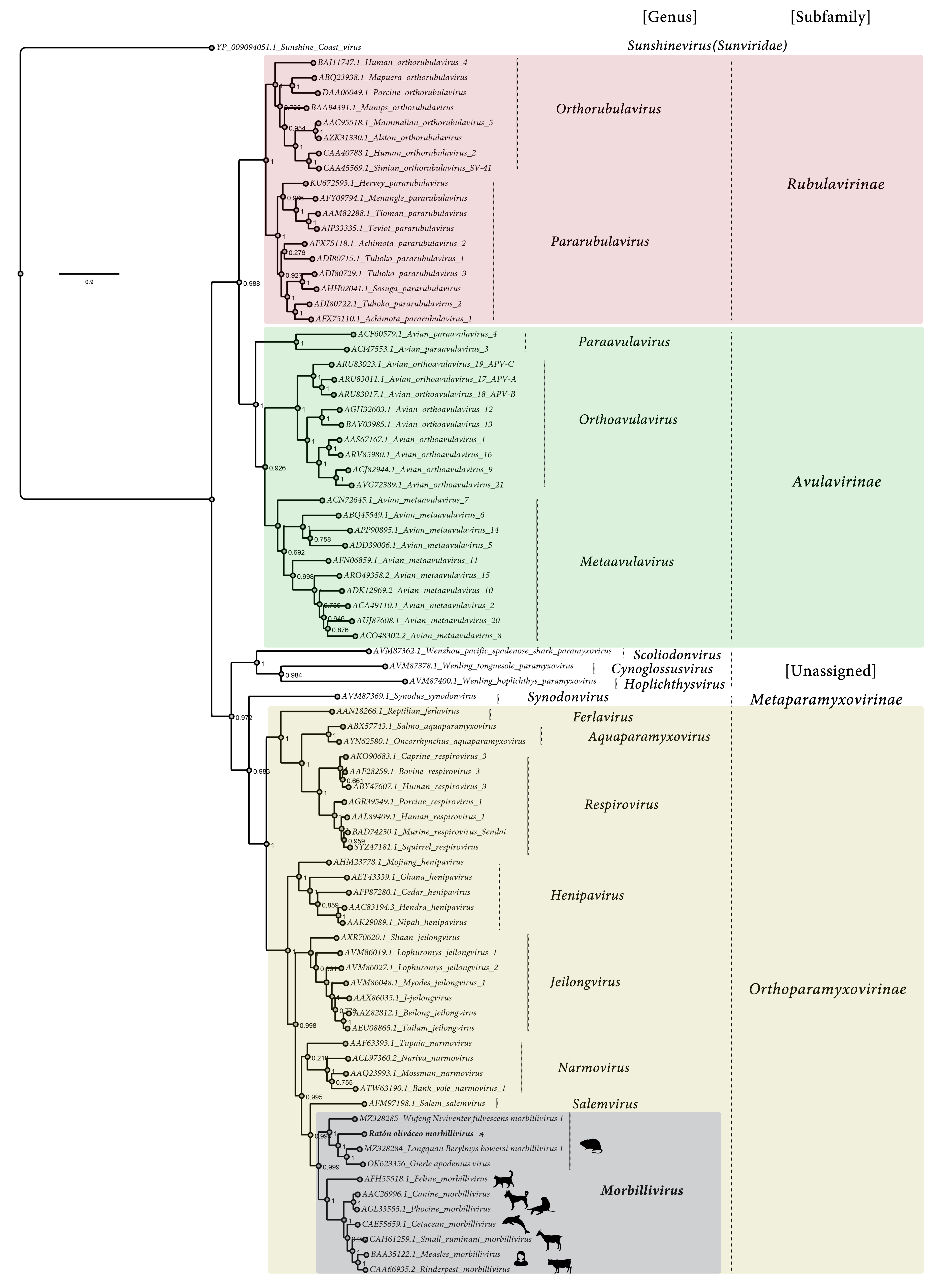

Supplement: Supplementary file 1 [file viruses-14-02403-s001.zip › Supplementary Figure S9.jpg]
